# Biometry and phenology of two sibling *Phylloscopus* warblers on their circum-Mediterranean migrations

**DOI:** 10.3897/zookeys.530.5955

**Published:** 2015-10-28

**Authors:** Piotr Zduniak, Reuven Yosef, Keith J. Bensusan, Charles E. Perez, Piotr Tryjanowski

**Affiliations:** 1 Department of Avian Biology and Ecology, Faculty of Biology, Adam Mickiewicz University, Umultowska 89, 61-614 Poznań, Poland; 2 Ben Gurion University − Eilat Campus, P. O. Box 272, Eilat 88000, Israel; 3 University of Gibraltar, Gibraltar Botanic Gardens Campus, P.O. Box 843, Gibraltar; 4 Gibraltar Ornithological and Natural History Society (GONHS), Jews’ Gate, Upper Rock Nature Reserve, P.O. 843, Gibraltar; 5 Institute of Zoology, Poznań University of Life Sciences, Wojska Polskiego 71 C, 60-625 Poznań, Poland

**Keywords:** geographical barrier, Mediterranean, migration, *Phylloscopus bonelli*, *Phylloscopus orientalis*

## Abstract

The Mediterranean Sea is known as an ecological barrier for numerous migratory birds flying from European breeding grounds to African wintering sites. Birds generally avoid migration over open sea and fly over land. In the Mediterranean Basin, few land bridges or bottlenecks for migratory birds exist. The narrowest are at the western and eastern extremes: the Strait of Gibraltar and Israel. Comparative studies between these locations are extremely rare to date. Therefore, in order to elucidate the differences between the two flyways, we compared data collected simultaneously for two sister leaf warbler species, the Bonelli’s Warbler complex, *Phylloscopus bonelli* and *Phylloscopus orientalis*, at ringing stations in the western Mediterranean Basin Gibraltar, and the eastern Eilat, Israel. Data on biometrics and passage dates of individuals trapped at Gibraltar and Eilat were used, and it was found that mean arrival date of Western Bonelli’s Warblers at Gibraltar was 15 days later than Eastern Bonelli’s Warblers at Eilat. Furthermore, Western Bonelli’s Warblers had shorter wings than Eastern Bonelli’s Warblers. On the other hand, birds in Eilat were in poorer body condition than individuals in Gibraltar. The comparison between geographically distant stop-over sites contributes to furthering our understanding of the development of migration strategies across ecological barriers in sibling species. Our study showed that populations that breed in southwestern Europe migrate through Gibraltar and winter in West Africa are able to accomplish migration in comparatively good body condition. This is in contrast to those that winter in East Africa, migrate through Israel and have to endure the combined challenge of crossing the Sahel, Sahara and Sinai deserts before reaching their breeding grounds across southeast Europe and southwest Asia. Hence, the discrepancies described between the western and the eastern flyway suggest that individuals in the west, in general, migrate shorter distances, have a physiologically less demanding crossing of the North African deserts and appear to stage before their crossing the Strait of Gibraltar, a privilege unavailable to the migrants of the eastern flyway.

## Introduction

Bird populations migrate annually from breeding sites to wintering localities and back. This phenomenon influences not only their proximate behavior and ecology, but also their ultimate evolutionary processes (Newton 2008, Pulido and Berthold 2010). Evolution, leading to speciation, may be especially strong if bird populations are divided into different migratory patches, wherein different breeding populations use varied patches to avoid ecological barriers during migration (Newton 2008, Chernetsov 2012). In the European avian migratory system, the Mediterranean Sea is a well known ecological barrier for numerous migratory birds that fly from their European breeding grounds to African wintering sites after the breeding season and return in the spring (e.g., Rubolini et al. 2002). Most birds try to avoid migration over large expanses of water if possible and many choose to fly over land even if this results in an extended migration (e.g., Newton 2008, Chernetsov 2012, Zduniak and Yosef 2012). Alerstam (2001) evaluated a number of observed and potential detours in relation to the general predictions of maximum detours and found that reduction of fuel transport costs may well be a factor of widespread importance at wide ecological barriers (seas, deserts, etc). However, to date this hypothesis has not been tested using a study system of sister species migrating through different, geographically distant stop-over sites. Hence, we undertook a study at two extreme points of circumnavigation around the Mediterranean Basin, where birds migrate past Gibraltar in the west and through Israel in the east. Both are well-known bottlenecks for migratory bird populations that breed in Eurasia and winter in Africa (Finlayson 1992, Yosef and Tryjanowski 2002, Partida 2006, Bensusan et al. 2007, Newton 2008, Zduniak and Yosef 2012).

Intra- and interspecific comparisons of the two bottlenecks are of interest because they are located at extreme ends of the Mediterranean Basin and birds use them to avoid long sea crossings. However, the two sites are ecologically distinct: Gibraltar is located on a mountain range with well-developed vegetation and has a hinterland with typically Mediterranean vegetation whilst Eilat is situated in the midst of a complex of deserts (Sahara to the south, Arabian to the east, Sinai to the west, Negev to the north). Differences in habitat around stop-over sites may favor different migratory strategies, especially phenology and time of stop-over use (Jakubas and Wojczulanis-Jakubas 2010, Kovács et al. 2011).

Leaf warblers (*Phylloscopus* spp.) are considered model species in bird migration studies because of their wide spectrum of ecological and migratory traits. Nevertheless European studies are mostly limited to Willow Warbler (*Phylloscopus trochilus* Linnaeus, 1758) and Chiffchaff (*Phylloscopus collybita* Vieillot, 1817) (e.g., Norman 1983, Green 1988, Catry 2005, Barriocanal and Robson 2007) and present knowledge of leaf warbler migration is incomplete (Ciach 2009).

In this paper, we analyzed two sibling leaf warbler species: the Western and Eastern Bonelli’s Warbler (*Phylloscopus bonelli* aVieillot, 1819 and *Phylloscopus orientalis* Brehm, 1855). These species are biogeographically and ecologically separated and were recently split as full species, having previously been classified as subspecies of *Phylloscopus bonelli* (Helbig et al. 1995, Baker 1997, Sangster et al. 2002). The Western Bonelli’s Warbler breeds in southwest Europe and North Africa and the Eastern Bonelli’s Warbler in southeast Europe and Asia Minor (see Marchetti et al. 1995, Katti and Price 2003). The species also have separate wintering ranges. The Western Bonelli’s Warbler winters in a narrow belt along the southern edge of the Sahara, mostly 10–17°N, from Senegal and southern Mauritania east to the Lake Chad basin. The winter quarters of the Eastern Bonelli’s Warbler are in northeast Africa (Sudan and Ethiopia), with some perhaps further west (Cramp 1998).

In order to avoid migration over large expanses of desert and then water, both species have to migrate over the Sahara and then circumnavigate the Mediterranean basin, crossing at Gibraltar in the west and Israel in the east. Because information in many handbooks and ringers manuals is, to date, strongly limited for both species and not provided to the species level (given as races/subspecies), basic morphometric and phenology studies are considered to be of importance (*cf.* Moore and Kerlinger 1987). Moreover, information regarding the migration of these sibling species at such migratory hotspots is scarce. Hence, the aim of the study is to describe differences in phenology at two stopover sites located on the threshold of the ecological barrier that stretches across the breadth of North Africa and to check for biometric differences between two sibling species of leaf warblers. We also hoped to further our understanding of the (dis-)similarities in the migration strategies of two sibling species with different wintering grounds, migratory routes with different geophysical barriers, and breeding areas. The two stopover sites are characterized by different habitat types and positions relative to the extensive desert barriers: Gibraltar is separated from desert habitats by hundreds of kilometres whilst Eilat is surrounded by it on all sides. This probably reflects their quality and importance for migrants.

## Methods

### Study areas

Owing to the fact that bird migration in the Mediterranean Basin is North-South-North, we collated data simultaneously at the two outermost sites (east and west), located ca. 3820 km from each other.

The Rock of Gibraltar is located at the eastern end of the Strait of Gibraltar (36°07'N; 5°21'W), where the Mediterranean meets the Atlantic. It lies ca. 21 km north of North Africa. Habitat in Gibraltar is typically Mediterranean and the vegetation around the ringing station at Jews’ Gate consists of dense maquis that is dominated by fruit-bearing shrubs (Perez and Bensusan 2005). Gibraltar is separated from Morocco’s arid, desert type ecosystems by ca. 400 km.

Eilat (29°33'N; 34°57'E) is located at the southernmost end of Israel and at the northern tip of the Gulf of Aqaba. Eilat provides a land bridge between three continents, Africa, Asia and Europe, and is located along a set of flyways used by birds wintering in sub-Saharan Africa and breeding in Eurasia. For migratory birds, Eilat is located before (late summer and autumn, or post-nuptial migration) or after (spring, or pre-nuptial migration) the Sahel, Sahara and Sinai Desert crossings, at the edge of almost 2000 km of continuous desert. To the northeast lies the Syrian Desert and to the east the Arabian Desert (Yosef 1997). Directly to the north is the Negev Desert (Izhaki and Maitav 1998).

### Field methods

Data analyzed were collected during the same period of 19 years, from 1992 to 2010. All trapping at Gibraltar was undertaken with mist-nets which were operated for ca. 6 hrs daily during the spring (March-May) and autumn (September-November) migration seasons. At Eilat, birds were trapped with mist nets for ca. 6 hr/d in the years 1992–1999. After 2000, birds were trapped using eight Helgoland/Rybachy traps, located along the trapping lanes of the mist-nets in the boundaries of the Eilat Bird Sanctuary and operated for ca. 6 hrs daily during the migration seasons. At both ringing stations, the trapping effort did not differ between spring and autumn in individual years and did not change throughout each season.

All trapped birds were ringed with the standard aluminum rings and biometrics were recorded. Flattened maximum wing chord was measured to the nearest millimeter and body mass determined using Pesola and/or digital scales to the nearest 0.1 g. Although adult *Phylloscopus* warblers have slightly longer wings than juveniles, as is the case in most passerines, we did not attempt to include age of birds in the analyses because leaf warblers are notoriously difficult to age during the pre-nuptial migration, following a complete molt in the wintering areas (Svensson 2006).

### Data processing and analysis

During the 19 years of study, 1101 Western Bonelli’s Warblers and 1706 Eastern Bonelli’s Warblers were ringed. Both species were recorded mainly during the pre-nuptial passage (98.1% and 99.2% of all ringed individuals of Western and Eastern Bonelli’s Warblers, respectively). Hence, owing to the sparse post-nuptial migration data we analyzed only data collected during the pre-nuptial migration. Furthermore, for the pre-nuptial period we chose to include in the analyses only those seasons for which at least ten individuals were recorded (18 springs in Gibraltar with insufficient data for 1999 and 16 springs in Eilat with insufficient data for 1994, 1997 and 2004). The average sample size per season was 59.6 birds (95% CL: 38.5–80.7, N = 18) for Gibraltar and 104.6 birds (95% CL: 58.0–151.3, N = 16) for Eilat. The total number of birds included in the in-depth analysis was 1073 for Gibraltar and 1674 for Eilat.

We checked for possible differences between species in migration phenology according to Julian days and the biometric parameters wing chord length and body mass. In these analyses only data from the first captures were used. Moreover, to examine possible differences between stopover sites, which have habitat types that probably differ in quality and importance for migrants, we also calculated and compared body condition index (body mass divided by wing length; e.g., Markovets et al. 2008, Zduniak and Yosef 2011, Zduniak and Yosef 2012) as well as the seasonal probability of recapture of ringed birds during each passage season. We considered recapture as that of individuals recorded subsequent to the day after first ringing, or later in the same migration season.

Because timing of migration and biometric parameters (wing chord length, body mass and body condition index) compared between species at the two study sites could be influenced by year, we controlled for year as a factor in the analyses. Comparisons between species were performed using Main Effects ANOVA with species and year as factors. Furthermore, we checked for patterns in changes of biometrics during spring migration. In the analyses we used a standardized time of migration for each study site calculated by subtracting the median Julian date of catching time for each spring season from each catching date in that season. Full biometric data were not always available for all individuals ringed and this has resulted in different sample sizes between individual analyses.

Standard statistical methods were used to describe and analyze the data (Zar 1999). All statistical tests were two-tailed. Calculations were performed using STATISTICA for Windows (StatSoft Inc. 2011). Throughout the text, mean values are presented with 95% confidence limits (CL).

## Results

### Phenology of migration

**Figure 1.**
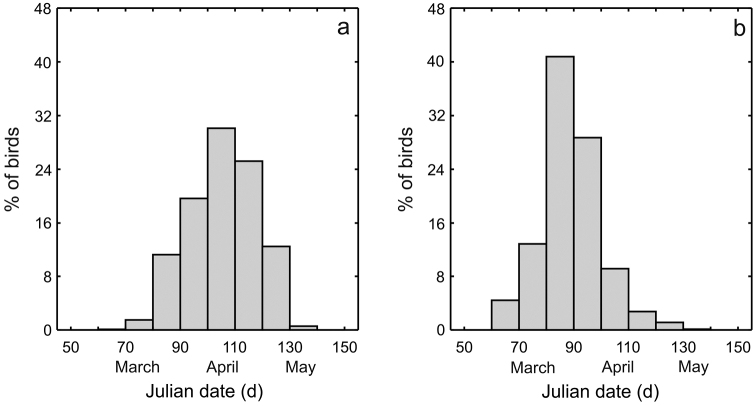
Migration phenology of Western Bonelli’s Warbler in Gibraltar (N = 1073; **a**) and Eastern (N = 1674; **b**) Bonelli’s Warbler in Eilat, Israel.

In general, migration period and duration of spring passage were similar for both species. Both in Gibraltar and Eilat the passage began in early March and lasted to mid May. However, the main passage period of Western Bonelli’s Warbler occurred from the third decade of March till the second decade of May with the peak at the second decade of April (Fig. 1a). The majority of Eastern Bonelli’s Warblers migrated between the second decade of March till the third decade of April and the peak of passage was at the third decade of March (Fig. 1b). Mean arrival date of Western Bonelli’s Warbler at Gibraltar was 15 days later than Eastern Bonelli’s Warbler at Eilat (two-way ANOVA, F_1,2727_=855.44, P < 0.001, x=105.5 day, CL: 104.7–106.2, range: 66−136 *vs.* x=90.3 day, CL: 89.6–91.1, range: 61−139, respectively).

### Wing chord length, body mass and body condition index

Western Bonelli’s Warblers trapped in Gibraltar had shorter wings (two-way ANOVA, F_1,2718_ = 352.73, P < 0.001; Table 1) and were slightly heavier (F_1,2651_ = 58.18, P < 0.001; Table 1) than Eastern Bonelli’s Warblers at Eilat. Furthermore, Western Bonelli’s Warblers were in significantly better condition than individuals recorded in Eilat (two-way ANOVA, F_1,2649_ = 289.75, P < 0.001; Table 1). The difference in body mass between individuals that had the same wing length was on average 0.5 g between sites. Taking into account the similar average body mass of populations of both species analyzed (Table 1), the difference averages to about 7% of body mass. Minimal and maximal values obtained for body mass and body condition index for both analyzed species were similar (Table 1). The exception was wing chord length, for which the range was greater for Eastern than for Western Bonelli’s Warbler (Table 1).

**Table 1.** Mean values with 95% confidence of limits in parentheses, ranges and sample size for wing chord length, body mass and body condition index of Western and Eastern Bonelli’s Warbler recorded in Gibraltar and Eilat, Israel, respectively.

Variable	Western Bonelli’s Warbler	Eastern Bonelli’s Warbler
wing chord length (mm)	63.6 (63.4−63.8) range: 56−73, N = 1072	66.0 (65.9−66.2) range: 49−77, N = 1666
body mass (g)	7.2 (7.2−7.3) range: 4.8−10.5, N = 1025	7.0 (6.9−7.0) range: 4.6−11.0, N = 1646
body condition index	0.114 (0.113−0.115) range: 0.075−0.161, N = 1025	0.106 (0.105−0.106) range: 0.073−0.167, N = 1644

A significant decrease of the wing length during the spring passage was recorded both in the case of the Western as well as the Eastern Bonelli’s Warbler (Pearson correlation, r = -0.28, N = 1072, P < 0.001; r = -0.29, N = 1666, P < 0.001, respectively). However the body condition of migrants decreased over the passage only in the case of Eastern Bonelli’s Warbler (r = -0.15, N = 1644, P <0.001) and no such pattern was observed in the case of Western Bonelli’s Warbler (r = -0.03, N = 1025, P = 0.323).

### Retraps

Throughout the study period we retrapped 17 (1.6%) Western and 339 (20.2%) Eastern Bonelli’s Warblers. The probability of Western and Eastern Bonelli’s Warblers recapture during the pre-nuptial period differed significantly between sites (Mann-Whitney U test, Z = 3.37, P < 0.001). The mean probability of recapture was 0.009 (CL: 0.020–0.017, N = 18) at Gibraltar and 0.159 (CL: 0.090–0.228, N = 16) at Eilat.

## Discussion

This study documents differences in migration phenology and energetic requirements between the Western and the Eastern Bonelli’s Warblers at the two extremes of the Mediterranean Sea: Gibraltar in the west and Eilat, Israel, in the east. The Eastern Bonelli’s Warbler peaked in the third decade of March, whereas the Western Bonelli’s Warbler peaked in the second decade of April; the mean arrival date of Western Bonelli’s Warbler at Gibraltar was 15 days later than that of Eastern Bonelli’s Warbler at Eilat. Western Bonelli’s Warblers trapped in Gibraltar had shorter wings and were in significantly better condition than Eastern Bonelli’s Warblers at Eilat, but the body condition of migrants only decreased over the passage period in Eastern Bonelli’s Warbler. Furthermore, a decrease of wing length was found during the spring passage in both species. Also, the mean probability of recapture was greater at Eilat.

Gibraltar and Israel (especially Eilat in the south) are located along very important flyways for birds migrating between Eurasia and Africa. Comparisons of data collected at such ringing stations can shed new light on bird migration strategies after having crossed the Sahara Desert and around the Mediterranean Sea. However, although ringing operations have been conducted at these two migratory hotspots simultaneously since 1992, a study comparing their respective data has never been undertaken. To our knowledge and in spite of numerous advances in migration research over the past decades (Newton 2008), the results presented here are the first encompassing the circum-Mediterranean avian migration system. It provides additional data regarding the phenomenon and highlights the importance of such comparisons.

A large disparity in numbers was recorded between the spring and autumn migration seasons at both sites. Almost all migrants from both species were recorded during the pre-nuptial passage (98.1% and 99.2% of all ringed individuals of Western Bonelli’s Warbler at Gibraltar and Eastern Bonelli’s Warbler at Eilat, respectively). A similar disparity has also been observed in some other species of long distance migrant passerines studied at Eilat (e.g., Yosef and Tryjanowski 2002, Yosef and Chernetsov 2004, 2005, Zduniak and Yosef 2012).

In the case of both ringing sites, the very low number of birds recorded during the post-nuptial migration could be the result of the majority of Western and Eastern Bonelli’s Warbler populations having their stopovers further to the north, allowing them to overfly Gibraltar and Eilat on their way south (e.g., Markovets et al. 2008, Zduniak and Yosef 2012). Certainly in the case of Gibraltar, important breeding grounds lie within close proximity and birds breeding in southern Iberia at least may not need to stop at Gibraltar so soon after commencing their migration. Such a strategy would also avoid potentially strong competition for food during a period in which, around the Strait of Gibraltar at least, availability of invertebrates is low (Bensusan 2007). However, one must also take into account that the Moroccan side of the Strait of Gibraltar has not been studied and it is not well understood whether some of the migrant populations stop over prior to initiating the desert crossing. Also, Western Bonelli’s Warblers breed in North Africa. This suggests that some of the European migrants may only have to cross the Strait of Gibraltar to find safe stopover sites, resulting in a short migratory hop.

It is also possible that both sibling-species engage in loop migration, wherein the majority of the population flies to the wintering grounds along a different flyway, or that both species migrate on a broad front on their way to Africa by flying straight across the Mediterranean Sea (Moreau 1961). However, upon their return journey both species have to cross the cumulative distance of about 2000 km of desert, which could shape the migration pattern further north. Many Western Bonelli’s Warblers are probably avoiding a long sea crossing by choosing the shortest route over the Mediterranean Sea - the Strait of Gibraltar - and are therefore recorded frequently at Gibraltar during spring. This is corroborated by Pilastro et al. (1998), who demonstrated the importance of the Strait of Gibraltar during the northward migration in their study of the migratory routes of eight trans-Saharan passerines through the central and western Mediterranean. From the 21 ringing points included in their study, Western Bonelli’s Warblers were most frequent at Gibraltar. In the case of Eastern Bonelli’s Warbler, the large number of migrants recorded in spring compared to the post-nuptial passage is most probably the result of the fact that the Eilat region is the first suitable staging area encountered after crossing an extensive desert. Therefore birds are forced to stop and refuel before continuing their northward migrations (*cf.* Tracy et al. 2005, Wojciechowski et al. 2005). This assumption is supported by the fact that within the same pre-nuptial migration seasons, mean probability of recapture of ringed individuals was 0.159 in Eilat and only 0.009 in Gibraltar. This indicates that Eilat is an important stopover site where Eastern Bonelli’s Warbler replenish their reserves just after the desert crossing and before they are able to continue to migrate north to their breeding grounds, whereas Gibraltar is located along the important northern flyway for Western Bonelli’s Warblers but is not so important for refueling. This is further confirmed by the difference in body condition index: Western Bonelli’s Warblers in Gibraltar were in better condition than Eastern Bonelli’s Warblers reaching Eilat. Once again, we raise the point of the need for future studies on the Moroccan side of the Mediterranean to understand the discrepancy. Furthermore, the data suggest that Eilat is used as a refuelling site by Eastern Bonelli’s Warblers because of their strenuous flight across the desert and the need to recoup physiologically (*cf.* Wojciechowski et al. 2014). This is strengthened by the fact that Chernetsov et al. (2007) found that Red-breasted Flycatcher (*Ficedula parva* Bechstein, 1794) caught in mist nets were significantly lighter in body mass than over-flying individuals that were shot, suggesting that weaker birds must stop to refuel while the healthier individuals overfly the site.

The morphometric data for the two taxa offered in the published literature are similar to this study. The mean wing length and body mass of Eastern Bonelli’s Warbler on a Greek island was 67.1 mm and 6.9 g, respectively (Barboutis et al. 2013). Similarly, the mean wing length and body mass recorded for Western Bonelli’s Warblers in north Morocco, Catalonia, the Balearics and the Columbretes were 60.8 mm−63.3 mm and 6.6 g−7.5 g, respectively (Gargallo et al. 2011). Mean wing length of Western Bonelli’s Warblers trapped in Gibraltar was shorter than that of Eastern Bonelli’s Warbler in Eilat (Table 1). Furthermore, the range of wing length was much wider for Eastern Bonelli’s Warbler than for Western Bonelli’s Warbler. The differences in wing length suggest that the geographic distance covered from the breeding to wintering grounds are not as large for the Western Bonelli’s Warbler as that of their Eastern sibling species, whose breeding distribution spreads from central Europe to central Asia. Another possible explanation is that a greater proportion of the birds caught at Gibraltar are juveniles (*cf.* Jenni and Winkler 1994). This could be particularly true if Gibraltar is not an important refueling site and landfall is incidental and more likely in less experienced birds. However, although the data remain speculative because of the unreliability of ageing both species, such a difference between sites seems unlikely, because a similar pattern between wing length and migratory period is observed at both sites.

Gwinner (1996) and Berthold (2001) thought that the beginning of the spring migration was controlled by the photoperiod or by endogenous programs initiated in the breeding grounds and winter in the sub-Saharan region. This appears to be true for both our study sibling-species, for which the migration period and duration of passage were similar when compared at the extreme points of the Mediterranean basin. Passage began in early March and lasted until mid May at both Gibraltar and Eilat. However, annual variations of spring migration phenology suggest that the initiation of the migration could also be connected to extrinsic factors (e.g., weather) in sub-Saharan Africa.

At Eilat, Eastern Bonelli’s Warblers peaked in the third decade of March. In contrast, for Eastern Bonelli’s Warblers on Antikythira, Greece, the median date of spring passage was 4 April (1^st^, 3^rd^ quartiles: 2 April, 14 April), with the earliest bird trapped on 21 March 2008 and the latest on 8 May 2010 (Barboutis et al. 2013). This indicates that Eastern Bonelli’s Warblers from the western extremes of its breeding distribution may be making a cross-Mediterranean flight later in the spring and that the populations migrating through Eilat are more Asiatic oriented. This was shown for migratory Chiffchaff at Eilat, where the species showed a strong directionality to the east with a very small proportion of only 13% towards the northwest (Ożarowska et al. 2004), even though all of the recoveries/controls from Chiffchaffs ringed at Eilat are from western and central Europe (Yosef 1997). A similar phenomenon might be occurring with the Eastern Bonelli’s Warblers, which could be spreading out like a fan after the desert crossing, towards their breeding grounds. This idea is further enhanced by the fact that Eastern Bonelli’s Warblers have been reported as common throughout Egypt between mid March and mid May (Goodman and Meininger 1989), unlike at Eilat. However, Horner (1977) found Eastern Bonelli’s Warblers mainly between 16 March and 12 April at Bahig on the northern coast, and the timing of migration in Egypt is in accordance with our results and substantiates the idea that the more western populations may undertake a sea crossing after the deserts.

Western Bonelli’s Warbler migration peaked in the second decade of April at Gibraltar. This is similar to Gargallo et al. (2011), who undertook a 16-year ringing project on western Mediterranean islands. They found that apart from the arrival of some very early individuals in mid-March, the main passage period occurred from late March onwards, with a peak at the end of April and a steep decrease thereafter, during May. This pattern was similar in all three study areas (N Morocco, Catalonia, Balearics, Columbretes). They contended that the overall, passage resembled the pattern described for birds on spring migration in the Gibraltar area (Finlayson 1992) and La Camargue (S France) (Blondel and Isenmann 1981). Along the Atlantic coast of Morocco, passage was somewhat earlier and was occasionally observed in late February in the south (Thévenot et al. 2003), although usually not until mid-March onwards in the SE (Gargallo et al. 2011).

It is of further interest that within the above mentioned similarity in migration period, the mean arrival date of Western Bonelli’s Warblers at Gibraltar was on average 15 days later than that of Eastern Bonelli’s Warblers at Eilat. One might argue that the position of Eilat at ca. 730 km further south than Gibraltar might play a part in this, although the discrepancy in the number of days is too great for the small difference in distance involved between the two sites. Differences may also be related to conditions in African wintering grounds, which can influence migratory patterns and phenology in particular, in a similar way to weather at stopover and breeding grounds (Gordo and Sanz 2006, Gordo 2007). However, a more plausible idea might be that inferred by combining the differences in wing chord length with phenology and inferring that the longer-winged Eastern Bonelli’s Warblers have a greater distance to cover to their breeding grounds, and hence arrive at Eilat *ca.* 15 days earlier than the Western Bonelli's Warblers, which have to fly a comparatively shorter distance.

We recommend future studies of similar focus to expand the scope by evaluating stop over sites on the Mediterranean coastline prior to their crossing the sea to Europe. This could explain why Isenmann (1989) recorded Western Bonelli’s Warblers migrating across France between the 11th of April and the 20th of May, later than at Gibraltar. Also, an effort must now be made by ringers to establish a method wherein monotypic species can be aged and sexed, either in the hand or by DNA-analyses. It is also of great interest to verify whether the migration routes assumed by researchers are indeed true in the field for the species. Orientation studies might show the azimuth of migration from North Africa and result in a fanning out of the Western Bonelli’s Warblers such that the westernmost populations of the species concentrate at Gibraltar, but those of the eastern extremities of their range are island-hopping across the western Mediterranean, as evidenced in the study of Gargallo et al. (2011).

Another point of interest in our data that relates to the idea forwarded that the two species may be fanning out of Africa to their breeding grounds in Europe, while the more easterly choose to stop over at Eilat, is the fact that we found a significant decrease in wing length during the spring passage in both study species. It has been established that longer distance migrants have relatively longer wings than conspecifics with shorter wings that migrate correspondingly shorter distances (for Eilat *cf.* Yosef and Meissner 2006, Yosef and Wineman 2010). It should be of interest to conduct an orientation, isotope analysis experiment to check if the shorter winged birds that arrive later in the season are also short distance migrants.

It is of interest that body condition of migrants only decreased over the passage period in the case of Eastern Bonelli’s Warbler and no that such pattern was evident in Western Bonelli’s Warbler. There may be several reasons for this loss of body mass. Older, more experienced birds are known to migrate more efficiently than inexperienced juveniles (*cf.* Yosef et al. 2006). However, our inability to age and sex the species prevents us from carrying out further analyses to understand if our assumption is correct. Another possibility is that conditions in sub-Saharan Africa do not allow the birds to better prepare for the crossing later in the spring. This could be further confounded by the inclement weather conditions that occur in the deserts in the late spring. This can only be verified by ringing birds in sub-Saharan Africa in the spring in a future study.

In conclusion, our results not only expand existing knowledge of leaf warbler *Phylloscopus* migration patterns (e.g., Ciach 2009), but also the observed similarities and differences in biometrics, migration phenology and ecology, which also validate further the recent taxonomic changes for these two *Phylloscopus* warblers. We were unable to corroborate Alerstam (2001), who considered energetic requirements and resulting stop-over sites to dictate migratory routes and strategies, owing to a lack of data of what is happening in Africa. Yet our study allows us to understand the relative importance of stop-over sites to migratory populations and the need for their continued conservation.
